# Does Threat Have an Advantage After All? – Proposing a Novel Experimental Design to Investigate the Advantages of Threat-Relevant Cues in Visual Processing

**DOI:** 10.3389/fpsyg.2019.02217

**Published:** 2019-09-27

**Authors:** Andras N. Zsido, Arpad Csatho, Andras Matuz, Diana Stecina, Akos Arato, Orsolya Inhof, Gergely Darnai

**Affiliations:** ^1^Institute of Psychology, University of Pécs, Pécs, Hungary; ^2^Department of Behavioural Sciences, University of Pécs Medical School, Pécs, Hungary; ^3^Department of Neurology, University of Pécs Medical School, Pécs, Hungary; ^4^MTA-PTE Clinical Neuroscience MR Research Group, Pécs, Hungary

**Keywords:** fear, visual search, perception, detection, evolutionary relevance, attention

## Abstract

The automatic visual attentional procession of threatening stimuli over non-threatening cues has long been a question. The so-called classical visual search task (VST) has quickly become the go-to paradigm to investigate this. However, the latest results showed that the confounding results could originate from the shortcomings of the VST. Thus, here we propose a novel approach to the behavioral testing of the threat superiority effect. We conducted two experiments using evolutionary relevant and modern real-life scenes (e.g., forest or street, respectively) as a background to improve ecological validity. Participants had to find different targets in different spatial positions (close to fovea or periphery) using a touch-screen monitor. In Experiment 1 participants had to find the two most often used exemplar of the evolutionary and modern threatening categories (snake and gun, respectively), or neutral objects of the same category. In Experiment 2 we used more exemplars of each category. All images used were controlled for possible confounding low-level visual features such as contrast, frequency, brightness, and image complexity. In Experiment 1, threatening targets were found faster compared to neutral cues irrespective of the evolutionary relevance. However, in Experiment 2, we did not find an advantage for threatening targets over neutral ones. In contrast, the type of background, and spatial position of the target only affected the detection of neutral targets. Our results might indicate that some stimuli indeed have an advantage in visual processing, however, they are not highlighted based on evolutionary relevance of negative valence alone, but rather through different associational mechanisms.

## Introduction

To this date, a large body of research has investigated whether there is automatic attentional processing that gives an advantage to threatening stimuli compared to non-threatening ones, using various paradigms. One of the first to studies on this field (Öhman, 1986; Davey and Dixon, 1996; Öhman and Soares, 1998) conducted the experiments using the Pavlovian conditioning paradigm. For instance, in a previous study (Öhman and Soares, 1998) participants were exposed to masked threatening (i.e., snakes and spiders) and non-threatening (i.e., flowers and mushrooms) pictures as conditioned stimuli, and mild electric shock as the unconditioned stimulus. Then, participants were shown the stimuli set, while their skin conductance was measured. They demonstrated unconscious conditioning (i.e., elevated SCR) only to threatening stimuli. However, further research from other laboratories using similar methodology failed to reproduce the same results (e.g., Menzies and Clarke, 1995; see Poulton and Menzies, 2002 for review), thus, this paradigm became widely considered less reliable.

Hence, Öhman et al. (2001) proposed a new methodology that uses an odd-one-out visual search task (VST). In this paradigm, participants are exposed to different numbers of pictures (typically 4 or 9) which are presented on a screen arranged in matrices (2 × 2 or 3 × 3, respectively). In half of the cases the pictures belong to the same category (e.g., flowers), in the other half, one picture differs from the others (e.g., a snake appeared among eight flowers). Participants have to decide whether all the pictures belong to the same category or there is a discrepant one. Their reactions were measured by pressing different keys. In the past decade, this paradigm became widely used in experiments with both adults (Blanchette, 2006; Soares et al., 2009a; Zsido et al., 2018b) and children (LoBue, 2010; LoBue et al., 2014; Zsido et al., 2018c) participants.

The advantage in visual processing for threatening stimuli has first been shown using this paradigm by Öhman et al. (2001), who later also coined the term *fear-module* (Öhman et al., 2001; Öhman and Mineka, 2003). They used pictures of evolutionary relevant cues, snakes and spiders as threatening and mushrooms and flowers as non-threatening stimuli. According to their results, participants responded to threatening stimuli faster than non-threatening ones. Plenty of research (X. Mineka and Öhman, 2002; Öhman and Mineka, 2003; Isbister and White, 2004; Soares et al., 2009a, 2017; Gao et al., 2017) showed that these animals – especially snakes (Soares et al., 2009b, 2014; Gomes et al., 2017) – had been present as human species evolved, and posed as a real threat to our species. This suggests that fast and accurate detection of potentially lethal animals might have been under positive selection in the evolutionary past (but cf. Al-Shawaf et al., 2019). The aforementioned studies used only evolutionary relevant cues. Therefore, in recent years, this theory was met with criticism (Flykt, 2005, 2006) because of the difficulty to generalize the findings to other types of threatening cues (e.g., modern).

Consequently, an alternative theory was proposed, namely, the *relevance superiority effect* (Sander et al., 2003, 2005; Fox et al., 2007; Subra et al., 2017; Zsido et al., 2018a, c). The relevance superiority effect suggests that people perceive fear-relevant – threatening in this context – stimuli faster than neutral ones regardless of evolutionary relevance. Brosch and Sharma (2005) conducted an experiment similar to the VST used by Öhman et al. (2001). However, they compared modern stimuli (e.g., gun, toaster) to evolutionary old ones (e.g., snake, flower). They concluded that in the case of fear-relevant stimuli – regardless of it being evolutionarily relevant or modern – participants recognized it faster compared to neutral ones. That is, the fear relevance of the cue seems to be more important than its evolutionary relevance (see e.g., H. Gao and Jia, 2017; March et al., 2017). Fox et al. (2007) suggested a similar conclusion based on a study, in which they found no difference in response to modern and evolutionary old fear-relevant stimuli. Nonetheless, previous studies used only a small number of exemplars per stimulus category – the best representatives (e.g., snake for evolutionary relevant, gun for modern threat) for the most part (see e.g., Zsido et al., 2018b for a collation).

Nearly a decade after the first research using the VST proposed by Öhman et al. (2001), LoBue and DeLoache (2008) introduced a change in the methodology. Instead of registering the different keypress responses, they used a touch-screen monitor to collect data from their participants. They claimed that responding with touch-screen monitor simplifies the task and responding itself and it takes out the use of a keyboard that can lead to several issues: (1) There is no need to use stimuli that do not contain a target picture, which was commonly used in previous experiments to reduce the possibility of response learning – but doubled the length of the task. Moreover, (2) there is no need to counterbalance hands that would otherwise lead to confounding results (due to collapsing responses of dominant and non-dominant hands). Further, (3) the lack of target-absent trials opened up the possibility to increase the repetitions of stimuli presentation or use more exemplars per stimulus category. LoBue and Matthews (2014) showed that the pattern of results is the same across the two types of responses, i.e., key press and touch-screen. Thus, we think further research should utilize touch-screen-based response collecting.

Nonetheless, the VST has been challenged (Rinck et al., 2005; Quinlan, 2013; Quinlan et al., 2017; Subra et al., 2017; Zsido et al., 2018a). After surveying a large body of literature Quinlan (2013) argued that it is unclear whether distractors or targets caused the results, i.e., the results are due to faster detection of the target, or a faster rejection of the distractors (see also Rinck et al., 2005; Fox et al., 2007). Furthermore, it was also shown (Subra et al., 2017) that since respondents need to process all the simultaneously presented pictures and select one of them, this task does not quite resemble the originally intended process of attention capturing. Finally, there is growing evidence (Cave and Batty, 2006; Notebaert et al., 2011; Quinlan, 2013) suggesting that controlling for potential perceptual confounds such as luminance, contrast and spatial frequency is crucial in studies dealing with VSTs.

A new approach emerged when studies started to use eye-tracking devices (Menneer et al., 2008; Humphrey et al., 2012; Godwin et al., 2015; Muhl-Richardson et al., 2018). For instance, Humphrey et al. (2012) created digital photographs depicting real-life scenes (e.g., street, bedroom) containing a target that could be emotionally charged (i.e., positive or negative) or neutral. Participants completed recall and recognition memory tests after seeing all the pictures; eye-movements were also recorded. Results showed that respondents recognized negative targets more accurately and recalled them in greater detail than positive and neutral ones. According to eye-movement data, participants spent more time fixating on negative targets than positive or neutral ones. Thus, Humphrey et al. (2012) concluded that negative emotional stimuli did have an advantage in attentional processing. Nonetheless, it is important to point out that the content of the negative target ranged from those provoking disgust to those depicting fear-relevant animals or object (e.g., snakes, knives). In our view, the methodology Humphrey et al. (2012) used provides a sound alternative to the VST and can be adapted into behavioral testing.

The overarching goal of our study was to introduce a novel paradigm that could eliminate the aforementioned shortcomings by (1) using both evolutionary relevant and modern cues with (2) more exemplars per stimulus category. Further, the current paradigm incorporates methodological innovations such that a (3) higher ecological validity and (4) utilizing touch-screen monitors, and thus, could serve as an alternative to the VST. The use of the present paradigm could help disentangle the confounding results and underlying factors of advantaged detection of threat. We sought to test whether evolutionary threatening cues have an advantage over neutral and modern threatening ones, or threatening cues regardless of evolutionary relevance have an advantage over neutral ones. In the first experiment, we used the most frequently included best prototype (see e.g., Fox et al., 2007; LoBue and DeLoache, 2008; Öhman et al., 2001) of the modern (gun) and evolutionary relevant (snake) threatening categories and compared them with neutral cues. Then, in the second experiment, we broaden these categories and included several more exemplars (i.e., knife, syringe, and spider, scorpion, respectively). We hypothesized that all threatening targets will be detected faster compared to neutral ones regardless of their evolutionary relevance.

## Experiment 1

### Methods

#### Participants

Thirty-four participants (16 men, 18 women) were recruited from students of the University of Pécs, with a mean age of 21.3 (S.D = 1.78). The sample size for this experiment was determined by computing estimated statistical power (β > 0.8, η^2^ = 0.15) based on the results of prior experiments on threat advantage (Öhman et al., 2001; Blanchette, 2006; Fox et al., 2007) using G^∗^Power 3 (Faul et al., 2007). All subjects were right-handed with normal or corrected-to-normal vision and participated on a voluntary basis. Data from one respondent were excluded because of failure to follow instruction. Our research was approved by the Hungarian United Ethical Review Committee for Research in Psychology (EPKEB) and was carried out in accordance with the Code of Ethics of the World Medical Association (Declaration of Helsinki). Written informed consent was obtained from all participants.

#### Materials

A set of pictures depicting real-life scenes (background images) and target objects were collected from the internet. Each target was added to the backgrounds with digital editing. Eight neutral background pictures of the same size (800 × 600 pixels) were collected from the Internet. Half of these pictures depicted natural scenes (e.g., forest, riverside), the other half depicted modern ones (e.g., street, factory). The background picture filled in the whole screen. We, then, collected four types of target pictures: evolutionary relevant threatening (snake) and non-threatening (cat), and modern threatening (gun) and non-threatening (pen). Each category included three different pictures of the object. All targets had the same size (50 × 50 pixels, i.e., visual angle 1.26°× 1.26°). None of the background pictures contained any similar objects, and neither humans nor animals were present on them.

To avoid the possible confounding effects of uncontrolled variance in low-level visual properties, we used the Spectrum, Histogram, and Intensity Normalization and Equalization (SHINE) toolbox for MATLAB (Willenbockel et al., 2010) to ensure that our background pictures were equivalent with respect to luminance, contrast, and spatial frequency. As a result, all the images used were grayscale.

The target pictures were placed on the background images with an equal distribution regarding their position and different types of objects. To avoid the possible bias that different targets placed on different places of the backgrounds would result in an unequal contrast of the targets, each target was then cut out (60 × 60 pixel squares) of the background and entered together in the SHINE toolbox to achieve equal luminance, contrast and spatial frequency values. These images were then placed back on the backgrounds. The 10-pixel difference between the cut-out squares and the actual size of the targets allowed us to apply a gradually fading filter to mask the visual differences between the replaced images and the background.

**FIGURE 1:**
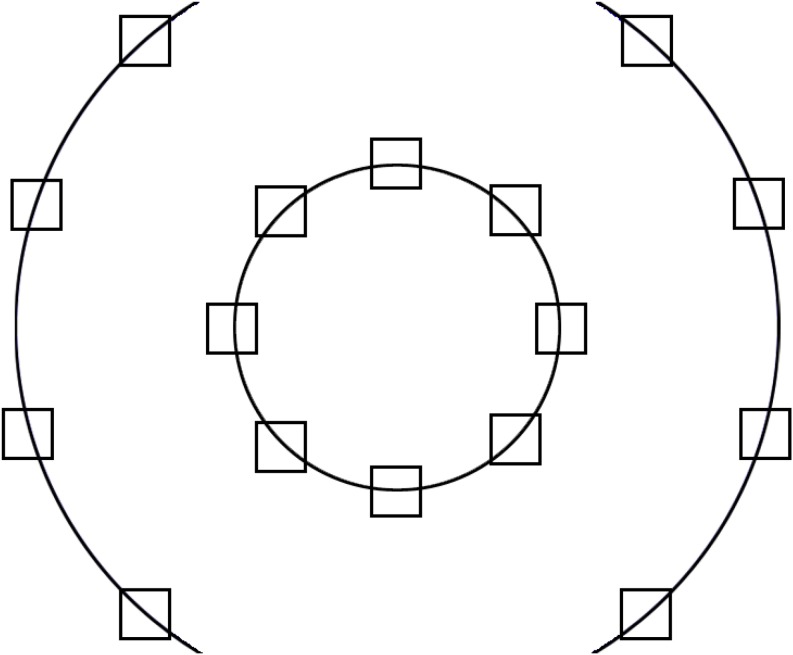
The 16 possible locations of the targets: 8 on the inner and 8 on the outer circle.

**FIGURE 2:**
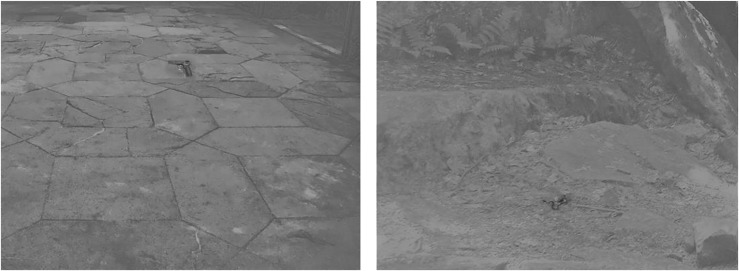
Examples of stimuli used. From **left** to **right**: A modern target on a modern background, and an evolutionary relevant target on an evolutionary background.

Images were presented on one of 16 possible locations of the screen (see Figure 1 for the layout). The image locations were distributed on two circles - and inner and an outer circle. The inner circle had a diameter of 400 pixels (10.08°), and the middle of the target pictures was placed on this with a distance of 157.08 pixels (3.97°). The diameter of the outer circle was 945 pixels (23.54°), to maximize the area covered. The distance between the targets on each side was 156.03 pixels (3.94°). Finally, we created the stimuli set that consisted of 16 (possible locations) × 8 (backgrounds images), i.e., 128 images. See Figure 2 for exemplars of the final stimuli set.

Image complexity was also calculated for all the final pictures, to control for the possible confounding effects of variance in visual complexity. The method we used is based on the assumption that average complexity increases as a function of log JPG file size (Donderi and McFadden, 2005; Forsythe et al., 2011). The pictures used in this experiment did not differ on this measure (*F*s < 1, *p* > 0.1).

#### Apparatus

The stimuli were presented on a 17-inch LG Flatron T1710 touch-screen color monitor, with a resolution of 800 × 600, 5:4 aspect ratio, refresh rate of 60 Hz, and color depth of 16.7 M. The gamma parameter was set to 1 in accordance with the SHINE toolbox recommendation to establish a linear relationship between the stored luminance of the image matrix and the luminance intensity of the screen. PsychoPy Software version 1.83 for Windows (Peirce, 2007) was used to present the stimuli.

#### Procedure

**FIGURE 3:**
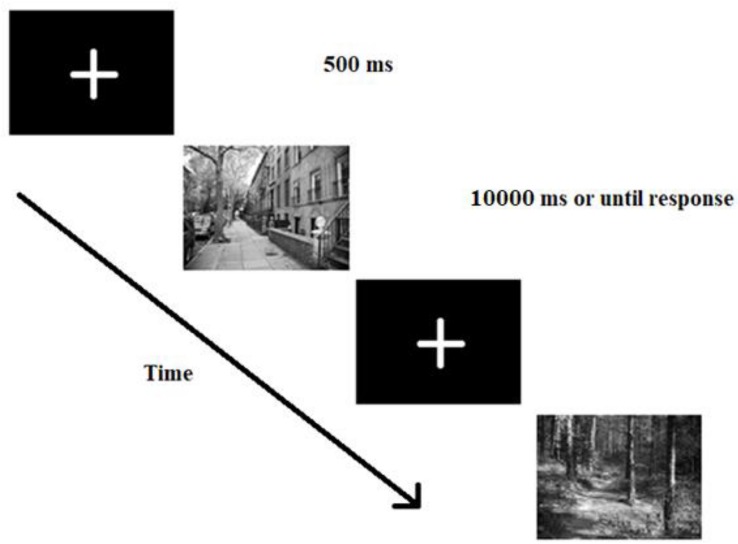
Trial presentation sequence used in both experiments. First, participants saw a fixation cross for 500 ms, then a real-life scene appeared with one of the target objects on it. Participants’ task was to find the target as quickly as possible and indicate the target’s location using a touch-screen monitor.

Participants were seated at a distance of approximately 60 centimeters from the monitor. First, subjects were shown the target stimuli used in the experiment and also asked to indicate how threatening they find each stimulus on a 7-point Likert-type scale (1 – not threatening at all to 7 – extremely threatening). See Supplementary Table S1 for central tendencies. This was done to make them familiar with the to-be found targets and to make sure the results are not due to different perceived threat level of the stimuli. Follow-up analyses confirmed that the gun and the snake were rated equally threatening (*t* < 1, *p* > 0.1) and threatening stimuli were rated more threatening than neutral ones (*F* > 2, *p* < 0.01). After the rating, participants were provided both with written and verbal task instructions. They were asked to respond with their dominant hand by touching the target on the screen and try to be as quick and accurate as possible. After responding, they had to place their hands on a paper in front of them on the table. Every trial began with a central fixation cross presented for 500 ms. The stimulus was shown for 10000 ms or until response. See Figure 3 for stimulus presentation sequence.

First, a set of 16 practice trials were given to teach respondents how to use the touch-screen monitor and, again, to get familiar with the pictures they were going to see. Practice trials were excluded from the data processing. After the practice session, the experimental session followed. Stimuli were presented in a randomized order. Response times and the spatial coordinates of the responses were recorded. Overall, the experiment took approximately 15 min.

### Results

First, we searched for inaccurate responses, when participants failed to touch the target on the screen. We did this using an outlier flagging procedure with a two-standard-deviation criterion on the raw data of coordinates. Response time data were deleted in these cases (<1%) and were considered as system missing during further analyses.

Trials with very low pointing accuracy (i.e., above the two-standard-deviation criterion on the raw data of coordinates) were excluded from the further analyses. This affected less than 1 per cent of the trials. None of the response times deviated from the normal distribution, neither the assumption of variances nor sphericity were violated. The statistical analyses were performed using the JASP Statistics Program (Version 0.8.6 for Windows).

We used a 2 × 2 × 2 × 2 repeated measures ANOVA to analyze the dataset with the Background (evolutionary relevant vs. modern), Origin of stimuli (evolutionary relevant vs. modern), Type of stimuli (threatening vs. neutral), and Position (inner vs. outer circle) being the fixed factors. See Table 1 for mean times and standard deviations for all categories.

**TABLE 1:** Mean response times and standard deviations in seconds for each category.

**Type of stimuli**	**Origin of stimuli**	**Background**	**Position**	**Mean**	***SD***
Threatening	Evolutionary	Evolutionary	Outer	1.437	0.184
			Inner	1.341	0.128
		Modern	Outer	1.457	0.141
			Inner	1.341	0.125
	Modern	Evolutionary	Outer	1.465	0.177
			Inner	1.325	0.107
		Modern	Outer	1.466	0.156
			Inner	1.341	0.117
Neutral	Evolutionary	Evolutionary	Outer	1.451	0.153
			Inner	1.475	0.289
		Modern	Outer	1.446	0.154
			Inner	1.340	0.105
	Modern	Evolutionary	Outer	1.457	0.189
			Inner	1.345	0.125
		Modern	Outer	1.529	0.187
			Inner	1.353	0.125

**FIGURE 4:**
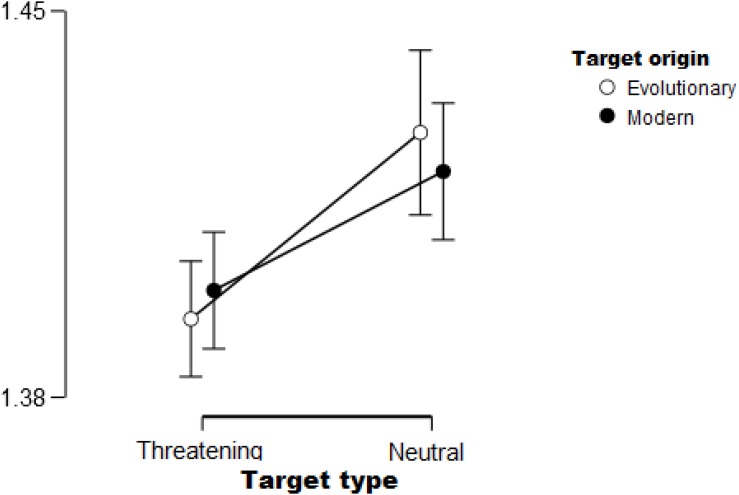
Threatening targets (snake and gun) were found faster compared to neutral cues (cat and pen), irrespective of target origin (evolutionary relevant or modern). Error bars (95% confidence interval) are shown.

As expected, the Type of the stimuli yielded a significant effect [*F*(1,33) = 8.57, *p* < 0.01, η*_*p*_*^2^ = 0.21]. Consistent with our hypothesis, participants were faster to find threat-relevant stimuli (snake and gun) than neutral ones (cat and pen). See Figure 4 for the main effect. The Position of the target was also significant [*F*(1,33) = 57.93, *p* < 0.01, η*_*p*_*^2^ = 0.64] such that targets on the inner circle were found faster than those presented on the outer circle. The Background and Origin of stimuli did not show a significant main effect (*F*s < 2, *p* > 0.1).

We found a Background × Target origin interaction [*F*(1,33) = 8.65, *p* < 0.01, η*_*p*_*^2^ = 0.21]; evolutionary relevant compared to modern targets were found faster on modern background and vice versa. Interestingly, both the Target origin × Position [*F*(1,33) = 19.04, *p* < 0.01, η*_*p*_*^2^ = 0.37] and the Background × Position [*F*(1,33) = 7.83, *p* < 0.01, η*_*p*_*^2^ = 0.19] interactions were significant: Participants found modern targets faster than evolutionary relevant on the inner circle, and vice versa on the outer one. Moreover, they responded to targets on the inner circle faster if they were present on a modern background, compared to evolutionary relevant; and vice versa on the outer circle.

The analysis also revealed three three-way interactions between Target type × Target origin × Background [*F*(1,33) = 9.61, *p* < 0.01, η*_*p*_*^2^ = 0.23], Target type × Target origin × Position [*F*(1,33) = 4.88, *p* < 0.01, η*_*p*_*^2^ = 0.13], and Target type × Background × Position [*F*(1,33) = 7.27, *p* < 0.01, η*_*p*_*^2^ = 0.18]. Interestingly, these interactions revealed that neutral targets were accountable for all three two-way interactions described previously.

The first interaction revealed that respondents found the evolutionary relevant neutral targets faster than modern ones on modern backgrounds and vice versa on evolutionary backgrounds [*F*(1,33) = 12.78, *p* < 0.01, η*_*p*_*^2^ = 0.28]. However, this effect was not present regarding threatening targets, as they were found equally fast irrespective of the type of the background [*F*(1,33) = 0.01, *p* > 0.1, η*_*p*_*^2^ < 0.01]. Neutral modern targets were found faster in the inner circle compared to the outer one [*F*(1,33) = 16.41, *p* < 0.01, η*_*p*_*^2^ = 0.33]; while this effect was not significant for the neutral evolutionary cues [*F*(1,33) = 2.82, *p* > 0.1, η*_*p*_*^2^ = 0.08]. Finally, neutral cues were found faster when placed on the inner circle compared to the outer one on modern background [*F*(1,33) = 50.96, *p* < 0.01, η*_*p*_*^2^ = 0.61], while they were found equally fast when presented on evolutionary relevant background [*F*(1,33) = 2.54, *p* > 0.1, η*_*p*_*^2^ = 0.07]. Threatening targets were always found faster when present on the inner circle irrespective of target and background type.

### Discussion

In our first experiment, we used the best prototype of the modern and evolutionary relevant threat categories and compared them to neutral stimuli using a new behavioral paradigm. The results showed that threatening targets were found faster than neutral ones, regardless of their origin (i.e., evolutionary or modern). Moreover, the significant effect of position underscores the importance of controlling for the eccentricity (Csathó et al., 2008) when presenting a stimulus, i.e., distance of targets from the fixation cross, in future experiments.

The interactions show that there are specific effects that are present for neutral stimuli but not for threatening ones. This might indicate that threatening cues are indeed preattentive (Öhman et al., 2001; Arnaudova et al., 2017) in the sense that they are more resistant to the context they are presented in. In contrast, it seems to be plausible to conclude that there is a *context effect* (Young et al., 2012) for neutral cues, i.e., the origin of the context determines whether evolutionary or modern targets are found faster. The results also suggest that targets are more salient when they are presented on a non-matching background.

Taken the above findings together, it seems to be that the confound and mixed results of previous experiments using the VST, as previous research (Quinlan, 2013; Quinlan et al., 2017; Subra et al., 2017) pointed out, could possibly originate from a pop-out effect, i.e., the interaction between the context and different subtypes of target stimuli. In conclusion, the results of our first experiment suggest a relevance superiority effect (Sander et al., 2003, 2005; Fox et al., 2007; Brown et al., 2010; Subra et al., 2017; Zsido et al., 2018a) over the evolutionary fear-module (Öhman et al., 2001; Mineka and Öhman, 2002; Öhman and Mineka, 2003), i.e., threatening targets are found faster compared to neutral targets regardless of their evolutionary relevance. Nevertheless, it must be noted that the current behavioral results say little about the underlying mechanisms that might be different for the evolutionary and modern threatening stimuli (Fang et al., 2016; Zsido et al., 2018c).

The motivation behind conducting a second experiment was to test whether the main effect of threat shown in Experiment 1 could be generalized to all threatening cues or it is specific to the most frequently used prototypes (i.e., snake and gun).

## Experiment 2

### Methods

Experiment 2 was similar to Experiment 1 in every aspect but one: we included more types of targets in both the evolutionary relevant and modern categories.

#### Participants

A new group of participants (15 men, 18 women) were recruited from students of University of Pécs, with a mean age of 22.1 (S.D = 1.59), matching the sample size of Experiment 1. All participants were right-handed with normal or corrected-to-normal vision. Data from two respondents were excluded because of failure to follow instruction. Our research was approved by the Hungarian United Ethical Review Committee for Research in Psychology (EPKEB) and was carried out in accordance with the Code of Ethics of the World Medical Association (Declaration of Helsinki). Written informed consent was obtained from all participants.

#### Materials

Again, all pictures used in this experiment were sourced from the Internet. We collected 24 background images (800 × 600 pixels) in total (including the previously used 8 from Experiment 1), half of it depicting evolutionary relevant scenes (e.g., grassland, forest) and the other half modern ones (e.g., side-walk, terrace). Four types of target categories were used: evolutionary relevant threatening (snake, spider, and scorpion) and non-threatening (cat, bird, and turtle), and modern threatening (gun, knife, and syringe) and non-threatening (pen, flashlight, and toaster). We selected these exemplars based on the stimuli used in previous studies (Blanchette, 2006; LoBue, 2010; Guerrero and Calvillo, 2016). Three different exemplars were used for all types of the objects (e.g., three snakes, three guns, etc.). All targets had the same size (50 × 50 pixels, i.e., visual angle 1.26°× 1.26°). None of the background pictures contained any similar objects, and neither humans nor animals were visible in the pictures.

Preparation of the final image set was identical to that of described in Experiment 1. Background images were modified by the low-level feature averaging technique using SHINE toolbox (Willenbockel et al., 2010). Then, targets were converted into grayscale, placed on the background images using the outer and inner circle layout as seen in Figure 1. Afterward, targets were cut out (60 × 60 pixels) and averaged on low-level visual features, and then placed back to the backgrounds. A filter was applied on the 10-pixel wide frame of the 50 × 50 pixel target to gradually fade the replaced image back to the background. The final stimuli set consisted of 16 (possible locations) × 24 (number of background images), i.e., 384 pictures.

We, again, calculated image complexity for all of the final pictures, with the same method used in Experiment 1 (Donderi and McFadden, 2005; Forsythe et al., 2011). The pictures used in this experiment were, again, similar in visual complexity (*F* < 1, *p* > 0.1).

#### Apparatus

Same as in Experiment 1.

#### Procedure

The procedure was identical to that described in Experiment 1. Similarly to Experiment 1, subjects were asked to rate how threatening they find each stimulus used in the experiment on a 7-point Likert-type scale (1 – not threatening at all to 7 – extremely threatening). See Supplementary Table S1 for central tendencies. Follow-up analyses confirmed that the modern and evolutionary threats were rated equally threatening (*F* < 2, *p* > 0.1) and threatening targets were rated more threatening than neutral ones (*F* > 2, *p* < 0.01). In sum, the experiment took for approximately 25 min.

### Results

First, similarly to Experiment 1, inaccurate responses were excluded using an outlier flagging procedure with a two-standard-deviation criterion on the raw data of coordinates. This affected less than 1% of the trials. The three subtypes of each target category were averaged resulting in the same 16 variables as in Experiment 1. None of these variables deviated from the normal distribution, and neither the assumption of variances nor sphericity were violated. The statistical analyses were performed using the JASP Statistics Program (Version 0.7.5 for Windows).

We had four factors, each with two levels: the Background (evolutionary relevant vs. modern), Origin of stimuli (evolutionary relevant vs. modern), Type of stimuli (threatening vs. neutral), and Position (inner vs. outer circle) being the fixed factors. Therefore, a 2 × 2 × 2 × 2 repeated-measures ANOVA was used to analyze the dataset. See Table 2 for descriptive statistics of response times.

**TABLE 2:** Mean response times and standard deviations in seconds for each category.

**Type of stimulus**	**Origin of stimulus**	**Background**	**Position**	**Mean**	***SD***
Threatening	Evolutionary	Evolutionary	Inner	1.307	0.127
			Outer	1.409	0.202
		Modern	Inner	1.307	0.155
			Outer	1.463	0.227
	Modern	Evolutionary	Inner	1.323	0.158
			Outer	1.433	0.239
		Modern	Inner	1.314	0.168
			Outer	1.447	0.180
Neutral	Evolutionary	Evolutionary	Inner	1.350	0.308
			Outer	1.419	0.232
		Modern	Inner	1.319	0.187
			Outer	1.409	0.200
	Modern	Evolutionary	Inner	1.301	0.156
			Outer	1.426	0.236
		Modern	Inner	1.316	0.172
			Outer	1.469	0.224

**FIGURE 5:**
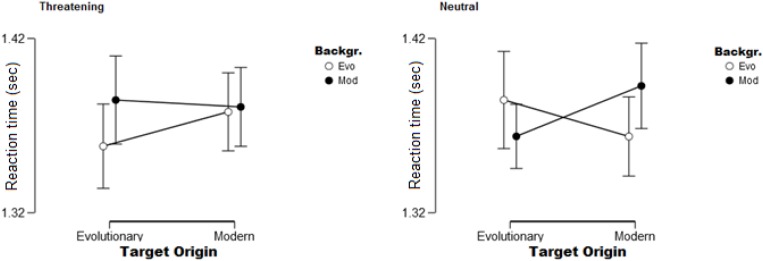
Significant Origin of stimuli × Background × Type of stimuli interaction. On the **left**: Origin of stimuli × Background interaction for threatening targets shows an advantage for evolutionary relevant cues present on the evolutionary background (Evo) compared to when present on the modern background (Mod). On the **right**: Origin × Background interaction for neutral targets shows a context effect, i.e., targets are found faster when presented on a background that has a different origin. Error bars (95% confidence interval) are shown.

Only one main effect, the effect of Position was significant [*F*(1,32) = 121.59, *p* < 0.01, η*_*p*_*^2^ = 0.79]. As expected, participants found targets faster on the inner position compared to when they appeared on the outer circle. Similarly to Experiment 1, we also found two significant two-way interactions: the Origin × Position of the target [*F*(1,32) = 10.67, *p* < 0.01, η*_*p*_*^2^ = 0.25], and Background × Position [*F*(1,32) = 11.45, *p* < 0.01, η*^2^* = 0.26] also reached significance. Modern targets were found slower on the outer circle compared to evolutionary ones; this effect, however, was not present when targets appeared in the inner positions. The same was found for the other interaction: targets were found slower on modern backgrounds when present in outer positions compared to evolutionary backgrounds; while they were found equally fast in inner positions regardless of the type of background. The analysis also yielded two significant three-way interactions. First, the significant Origin of stimuli × Position × Type of stimuli [*F*(1,32) = 7.12, *p* < 0.05, η*_*p*_*^2^ = 0.18] showed that the previously described effect regarding the Origin of stimuli × Position interaction is only true for neutral stimuli, and not for threatening cues. The other interaction is between Background × Origin of stimuli × Type of stimuli [*F*(1,32) = 6.53, *p* < 0.05, η*_*p*_*^2^ = 0.17] – see Figure 5. Interestingly, the Origin of stimuli × Background interaction is different for the two types of stimuli (threatening and neutral). Evolutionary relevant threatening targets seemed to have a slight advantage on evolutionary relevant backgrounds compared to when presented on modern backgrounds. While neutral targets showed a context effect, i.e., evolutionary relevant targets were found faster on modern backgrounds compared to evolutionary ones, and modern targets were found faster on evolutionary relevant backgrounds compared to modern ones.

### Discussion

In the second experiment, we compared four categories: evolutionary relevant and modern threatening stimuli, and evolutionary relevant and modern neutral cues. More representatives for each category were used compared to our first experiment in order to acquire a broader picture of the underlying mechanisms of threat detection. Interestingly, our results suggest that there is no difference in the detection of threatening and neutral cues, which is in accordance with the suggestion of some recent research (Horstmann, 2009; Quinlan, 2013; Yue and Quinlan, 2015).

In consent with Experiment 1, the effect of context works differently for threatening compared to neural targets, as indicated by the significant interaction. For threatening ones, the congruent background seems to help to find the target, but this effect is specific only to evolutionary relevant cues and not for modern ones. Contrary, for neutral targets, the incongruent background highlights the target and makes it easier to detect, and this effect works equally for evolutionary relevant and modern settings. This particular result might contribute to understanding the effects of the context the stimuli are presented (see also Vogt et al., 2011; Young et al., 2012; Gronau and Izoutcheev, 2017).

Regarding the position of the target, we found that neutral stimuli were found faster when presented on the periphery compared to when they appeared close to the middle of the screen. Threatening targets seem to be untouched by this effect. One possible explanation is that animate objects might have an advantage over inanimate ones in perceptual processing (see e.g., Sakaki et al., 2012). Furthermore, participants found targets faster on the outer compared to inner circle when they were presented on evolutionary relevant backgrounds, while they found targets faster on the inner compared to the outer circle on modern backgrounds. The two eccentricities used in the present study could also be seen as task difficulty (Csathó et al., 2008) with the inner circle being the easy and the outer circle being the harder one. Previous results (Li et al., 2002; Csathó et al., 2015) showed that the processing of evolutionary relevant natural scenes (similar to what we used as backgrounds) require minimal attentional capacity. Thus, we suggest that the interaction is due to the difference in the effort needed to process the background image, such that automatic processing made it possible to find the targets equally as fast in two eccentricities. The advantage was only measurable in the more difficult setting (i.e., outer circle) because the task used in our experiments was still relatively non-demanding. Nevertheless, we suggest that the position of the target stimuli should also be controlled in further studies.

## General Discussion

Our goal was to find clear evidence in the dispute over threat advantage. In two experiments, we test whether threatening targets have an advantage in visual detection, and if so, whether those with evolutionary relevance are highlighted in visual processing compared to modern cues. In Experiment 1, we used the best exemplars of the evolutionary relevant and modern categories, the snake and the gun, respectively. Our results suggest that there is an advantage for threatening cues over neutral ones, regardless of the evolutionary age. This supports the relevance superiority effect (see e.g., Fox et al., 2007; Subra et al., 2017). In Experiment 2, we repeated Experiment 1, but we used three different objects per category, to allow us to generalize the results of Experiment 1. Interestingly, however, we did not find a difference in visual search speed for threatening cues over neutral ones. This result by itself would suggest that there is no superiority effect for fear-relevant cues.

Nonetheless, we claim that the results of the two experiments mean that there are, indeed, specific threatening cues that have an advantage in visual search. Although this effect seems to be more specific, similar to the fear-module Öhman et al. (2001) suggested. It is possible that some stimuli are highlighted based on specific visual features (Wolfe et al., 1992; Coelho and Purkis, 2009), prepared learning or observation and verbally transmitted information (Seligman, 1971; LoBue and Rakison, 2013). Importantly, although the behavioral outcome is similar (i.e., fast reaction and quick detection) the underlying mechanisms might be different for stimuli with different evolutionary backgrounds (Fang et al., 2016; Zsido et al., 2018c). We argue that there is more than just fear-relevance. Snakes, for instance, often assume a characteristic pose with a curvilinear shape of their bodies. Based on the result of previous studies (Wolfe et al., 1992; LoBue, 2014; Van Strien et al., 2016) possibly the interplay of the curvilinear body shape and threat-relevance drive the facilitated response. Furthermore, even Öhman and Mineka (2001) pointed out that some modern threat-relevant stimuli (e.g., guns) could be detected as quickly as snakes in a VST because they are strongly associated with threat throughout the media, action movies and social interactions. Indeed, some stimuli might be more strongly associated with threat and fear, than others (Subra et al., 2017). Similarly as Seligman (1971) proposed regarding fear acquisition “*some contingencies are learned about much more readily than others*” (Seligman, 1971, pp. 313). We argue that some associations are not only learned more readily but also become stronger than others (see also Al-Shawaf et al., 2019). Thus, participants are more prone to answer quickly when such a stimulus is present in a VST.

In our view, the fact that the interactions between the origin of the background and the stimuli were specific to neutral targets suggest that threat itself has an advantage in processing as it is less sensitive to context, and other variables (Carretié et al., 2017; X. Gao et al., 2017). This effect has been shown consistently throughout both experiments. This novel finding may have further theoretical implications regarding the conceptualization of threat advantage. To this date, we found only one study (Young et al., 2012) to investigate contextual effects on threat detection. However, the authors used a priming paradigm and not a visual search of different targets in actual visual scenes like in our experiments. The unaffectedness by the context when detecting threatening targets could be explained by both attentional biases for threat (Fox et al., 2001; Cisler and Koster, 2010; McNally, 2018; Zsido et al., in press) and controlled attentional processing (Flykt, 2005; Flykt et al., 2007). Yet, this is similar to the encapsulated (cognitively impenetrable) nature of the fear-module (Öhman and Mineka, 2001). Taken together with the previous argument on various powers of stimulus association and threat, these results might point to different stages of advantaged visual processing of threats much like different stages of the guided search model by Wolfe (1994, 2007). Indeed, the number of specific threat-relevant characteristic (e.g., visual features, preparedness, the strength of association, etc.) a threatening stimulus has may determine at which stage it is processed. Thus, we suggest that although only a few specific threatening stimuli could elicit a distinctively quick behavioral response, all threatening cues are processed unaffected by the context they are presented in.

A particular strength of the experiments reported here is the exceptionally stringent controls used in the new paradigm. In most previous visual search studies, the low-level visual features were not equated, the target categories were not matched with all possible controls (e.g., only evolutionary or only modern categories used), moreover, they only used one or two exemplars (mainly snakes and guns). Our comparison of the detection of evolutionary relevant and modern, threatening and neutral targets provides strong evidence on the bias in the detection of some threatening targets.

Limitations of this study include the relatively small number of exemplars per target category (e.g., snakes, spiders, scorpions) used. This is a result of the relatively large number of categories, thus, the conclusions drawn from the results need further verification. Moreover, the repetitions we used could be further extended, however, the consequences of fatigue and the time-on-task effect should then be taken into account (Lim et al., 2010; Csathó et al., 2012). Follow-Up analysis on this matter showed a significant learning effect, but no fatigue effect. This means, that the repetition rate could yet be further extended. Further, in spite of our efforts to match the targets and backgrounds on low-level visual features, there are other variables that could cause confounding effects, for instance, the position of targets, visual array and familiarity of the backgrounds. Our efforts to match the low-level features inevitably resulted in gray-scale images that might reduce the ecological validity of our results. Finally, although previous studies in the past decade also used LCD monitors, a recent study showed (Zhang et al., 2018) that the time and spatial properties might differ between different modes of the LCD monitors, which may limit the comparability of our and previous results.

Despite these shortcomings, we showed that people are particularly prone to the rapid visual detection of some highlighted stimuli. These specific threatening cues, the snake and gun, seem to excel from others. This finding calls for a new theory that incorporates previous ones positing the existence of a bias to highly threatening cues that evolved during evolution or acquired through social connections during the ontogenesis.

## Data Availability Statement

The raw data supporting the conclusions of this manuscript will be made available by the authors, without undue reservation, to any qualified researcher.

## Ethics Statement

Our research was approved by the Hungarian United Ethical Review Committee for Research in Psychology (EPKEB) and was carried out in accordance with the Code of Ethics of the World Medical Association (Declaration of Helsinki). Informed consent was obtained from all participants.

## Author Contributions

AZ, DS, AA, and OI contributed to the conception and design of the study. DS and AA collected the data. AZ, DS, and AA organized the database and performed the research. AZ, OI, AM, AC, and GD performed the statistical analysis. AZ wrote the first draft of the manuscript. OI, AM, AC, and GD wrote sections of the manuscript. All authors contributed to the manuscript revision, read, and approved the submitted version.

## Conflict of Interest

The authors declare that the research was conducted in the absence of any commercial or financial relationships that could be construed as a potential conflict of interest.
