# Supplementary material for: Does Threat Have an Advantage After All? – Proposing a Novel Experimental Design to Investigate the Advantages of Threat-Relevant Cues in Visual Processing
Source: Front Psychol. 2019 Sep 27;10:2217. doi: 10.3389/fpsyg.2019.02217 (PMC6776589; doi:10.3389/fpsyg.2019.02217)
Supplement: Supplementary file 1 [file Table_1.docx]

**Supplementary Table 1** - Mean threat ratings (on a 7-pont Likert-type scale) and standard deviations of the targets used in Experiment 1 and 2

|  | **Experiment 1** | | **Experiment 2** | |
| --- | --- | --- | --- | --- |
| **Target** | **Threat rating** | | | |
|  | **M** | **SD** | **M** | **SD** |
| Bird | - | - | 1.07 | 0.26 |
| Cat | 1.12 | 0.49 | 1.04 | 0.52 |
| Turtle | - | - | 1.10 | 0.22 |
| Lamp | - | - | 1.08 | 0.19 |
| Pen | 1.11 | 0.23 | 1.03 | 0.22 |
| Toaster | - | - | 1.04 | 0.37 |
| Scorpion | - | - | 5.46 | 1.32 |
| Snake | 5.60 | 1.45 | 5.71 | 1.58 |
| Spider |  |  | 5.66 | 1.44 |
| Gun | 5.74 | 1.52 | 5.66 | 1.40 |
| Knife |  |  | 5.87 | 1.72 |
| Syringe | - | - | 5.33 | 1.65 |
